# Prevalence and Predictors of Seasonal Influenza Vaccine Uptake in Saudi Arabia Post COVID-19: A Web-Based Online Cross-Sectional Study

**DOI:** 10.3390/vaccines11020353

**Published:** 2023-02-03

**Authors:** Saeed Mastour Alshahrani, Yousef Zahrani

**Affiliations:** Department of Public Health, College of Applied Medical Sciences, King Khalid University, Khamis Mushait 62529, Saudi Arabia

**Keywords:** seasonal influenza, seasonal influenza vaccine, SIV, uptake, post COVID-19, prevalence, predictors, Saudi Arabia

## Abstract

In the fall of 2022, the number of influenza-like illnesses (ILIs) and severe acute respiratory infections (SARIs) in Saudi Arabia had significantly increased compared with the corresponding period in previous years. Concerns regarding the population’s seasonal influenza vaccine (SIV) uptake rates have emerged. In particular, the SIV uptake rates may have dropped post the COVID-19 pandemic compared with rates prior to the COVID-19 era. In this study, we aimed to estimate the prevalence and predictors of SIV uptake in Saudi Arabia post the COVID-19 pandemic. We conducted a cross-sectional study utilizing an online survey platform. We mainly collected sociodemographic information and determined whether the respondent was a healthcare professional or had a chronic disease. The overall SIV uptake prevalence was 31.8%. A lower SIV uptake was observed among those aged 55 years or older, females, residents of the central region, non-health practitioners, and those without chronic diseases. Several factors were associated with SIV uptake. Those aged 35–44 were over three-fold more likely to receive an SIV than those aged 55 years or older (OR: 3.66; 95% CI: 1.33–10.05). In addition, males had 73% higher odds of SIV uptake than females (OR: 1.73; 95% CI: 1.18–2.55). Health practitioners were more likely to receive an SIV than non-health practitioners (OR: 2.11; 95% CI: 1.45–3.06). Similarly, those with chronic diseases had 86% higher odds of SIV uptake than those without chronic diseases (OR: 1.86; 95% CI: 1.18–2.95). These findings can provide insights into the low prevalence and predictors of SIV uptake in Saudi Arabia. Future studies should be conducted to further explore the potential factors associated with such a low prevalence of SIV uptake post COVID-19 in Saudi Arabia.

## 1. Introduction

Influenza (flu) is a respiratory disease caused by multiple strains of the influenza RNA virus [1]. Based on the protein components, there are three main types of influenza virus: A, B, and C. Influenza type A is the most common type of flu virus, as it spreads in mammals—including humans—and birds [2,3]. The consistent changes in the genetic structure of the influenza virus throughout history have been responsible for various subtypes of influenza that have caused epidemics and pandemics, such as H5N1 and H1N1 [4]. The risk of other potential epidemics and pandemics due to influenza still exists because of the high transmissibility and severity of the infection [5].

Influenza and influenza-like illnesses have substantial health and economic impacts [6,7,8]. According to the World Health Organization (WHO), seasonal influenza is responsible for 650,000 deaths yearly because of respiratory diseases [9]. Approximately two-thirds of influenza-related deaths were among those aged 65 years or older [10]. In 2015, the economic burden of seasonal-influenza-associated healthcare services in the United States was estimated to be over USD 11 billion [11]. In China, the annual cost related to seasonal influenza was approximately 4 billion U.S. dollars [12]. The economic impact of seasonal influenza is primarily due to primary care services, hospitalizations, treatments, and flu-related absences from work [13]. A study from Italy found that the average number of absences due to influenza sick leave during the flu season was approximately 11,000 days/year [14].

Vaccination is the specific prevention measure for seasonal influenza and can also be used to control the spread of the infection [15]. The seasonal influenza vaccine (SIV) was first licensed in 1945 [16]. The vaccine has been recommended for the elderly and high-risk groups [17] and has effectively reduced the potential health and economic burden [18]. It is estimated that approximately USD 220 million of influenza-related costs every year can be avoided when a sufficient vaccine coverage rate is obtained [18]. In addition, the working days lost because of influenza-related absences were much lower among vaccinated individuals than those who were unvaccinated [19]. However, the low uptake rates of SIV remain a huge obstacle to achieving a greater positive impact on public health and the economy [20].

Low SIV uptake rates have been observed in multiple regions worldwide [21]. Despite the magnificent increase in SIV dose distribution between 2004 and 2011, due to a collaborative effort facilitated by the WHO, uptake rates have remained suboptimal [21]. Out of all of the SIV doses distributed globally, the number of doses distributed in regions including the Eastern Mediterranean, Southeast Asia, and Africa represented 3.7% [21]. In Europe, 44% of the targeted groups receives the SIV annually, whereas approximately 42% of the U.S. general population receives the SIV annually [21].

During the efforts to vaccinate against the COVID-19 pandemic, the previous history of SIV uptake strongly predicted COVID-19 vaccination [22,23]. On the other hand, previous COVID-19 vaccination was associated with a low intention to be vaccinated against seasonal influenza [24]. Low SIV uptake intention post the COVID-19 era was suggested to be due to mistrust of the healthcare system and the absence of perceived risk—as seasonal influenza is not lethal nor could it lead to severe complications or due to the fear of side effects [25,26]. 

In Saudi Arabia, the SIV uptake rates before the COVID-19 pandemic were 37.1% among those 65 years or older and 45% among a general population sample [27,28]. During the COVID-19 pandemic, a study conducted in northern Saudi Arabia found the prevalence of SIV uptake during 2021 was approximately 45% [29]. However, the study reported several reasons for people not taking the SIV, which included the fear of the interaction between SIV and the COVID-19 vaccine, and the excessive administration of multiple doses of the COVID-19 vaccine during the pandemic [29]. 

The Saudi Arabian health authorities reinforced multiple measures during the COVID-19 pandemic, which included offering COVID-19 vaccines free of charge for the entire population—including non-citizens—making vaccination compulsory for employees, and requiring completion of COVID-19 vaccine doses before traveling through airports [30]. These measures have been effective and helped mitigate the adverse impact of the pandemic [30]. The SIV is also free in Saudi Arabia for the entire population—including non-citizens. The Saudi Arabian Ministry of Health (MOH) has recently urged the population to visit primary healthcare centers (PHCs) to receive the SIV in response to the marked increase in influenza cases and influenza-like illnesses (ILIs) [31]. The Saudi Arabian MOH indicated that the number of influenza cases and ILIs during the flu seasons in 2022 was noticeably increased compared with the corresponding period during the last few years, implying a potential burden on the healthcare system in the Kingdom [32]. According to the WHO influenza surveillance reports, the percentage of positive influenza cases, the number of ILIs, and the number of severe acute respiratory infections (SARIs) have jumped from 3.75%, 45, and 82 in 2021 to 25.11%, 344, and 303 in the corresponding period of 2022, respectively [33]. The low incidence of ILI and SARI in 2021 can be attributed to the effective campaigns encouraging the co-administration of COVID-19 and seasonal influenza vaccination during the COVID-19 pandemic. On the contrary, the high ILI and SARI in 2022 can be attributed to low compliance with general preventive measures post COVID-19, but more crucially, to possible higher than usual hesitancy levels toward SIV. 

To the best of our knowledge, there are no recent reports in Saudi Arabia investigating the effects post COVID-19 on SIV uptake rates. Therefore, this study aimed to estimate the prevalence of the seasonal influenza uptake rate among the Saudi population, especially after the COVID-19 pandemic. In addition, we explored the factors associated with vaccine uptake. The results from this study should provide information to the Saudi MOH concerning targeting low-uptake groups, in addition to several recommendations in order to increase the uptake rates among the Saudi population.

## 2. Materials and Methods

### 2.1. Design and Participants

This cross-sectional study was conducted utilizing an online questionnaire distributed via social media, including Email, Twitter, and WhatsApp. The use of social media for data collection in health research has been suggested as a useful tool to reduce data collection costs and time and achieve better representation compared with the traditional recruitment method [34]. In order to reach a large sample of the Saudi population, the snowball sampling technique was used, as the person who had received the questionnaire was requested to resend it to their contacts. The questionnaire was sent across the five main regions of the Kingdom of Saudi Arabia. We restricted the inclusion to those who were aged 18 years or older who understood and spoke Arabic and resided in Saudi Arabia. The total sample included 758 participants.

### 2.2. Questionnaire

The questionnaire was developed in Arabic and reviewed by experts in the field for its coherence and for the face validity evaluation. Then, it was pre-tested using a small sample to ensure the clarity of the questions and choices. The questionnaire consisted of demographic information and medical history, including the presence of chronic disease, previous uptake of the SIV, and the time since their last vaccine was received. In addition, a question regarding whether the respondent was a health practitioner was included. 

### 2.3. Assessment of the Outcome

The primary outcome of this study was to determine whether the participant had received the seasonal influenza vaccine (SIV) within the preceding 12 months (yes; no). Specifically, we provided multiple choices for this question in order to precisely assess the time since their last vaccine was received (within the last three months, 3–6 months, and 6–12 months). We also added a question regarding whether the respondent had ever received the SIV. 

### 2.4. Assessment of the Predictors

The variables of interest investigated as potential predictors of seasonal influenza uptake were used as categorical variables (e.g., nominal, dichotomous, ordinal). These predictors included age group (18–24; 25–34; 35–44; 45–54; ≥55), gender (female; male), and education level (high school or less; bachelor’s degree or some college education; postgraduate), region of residence (central; southern; eastern; western; northern), being a health practitioner (yes; no), and having a chronic disease such as heart diseases, diabetes, hypertension, and hypercholesterolemia (yes; no). 

### 2.5. Statistical Analysis 

SPSS version 21.0 software (SPSS Inc., Chicago, IL, USA) was used to perform the analyses for this study. We reported descriptive statistics using frequency (n) and percentages (%). The prevalence of the SIV uptake was measured as the number of vaccine recipients divided by the total number of respondents and the number of vaccine recipients divided by the number of respondents in each subgroup of interest. We also used bivariate analysis between the covariates and the previous uptake of the SIV utilizing the chi-square test. We also used logistic regression to assess how each covariate could have predicted the uptake of the SIV. The assumptions of chi-square and logistic regression were explored; no violation was observed. We used α < 0.05 as the statistical level of significance for the current analyses.

## 3. Results

A total of 758 participants were included in the analysis. The majority of the participants were males (60.4%), aged 25–34 (32.6%), who had a bachelor’s degree or some college education (70.4%) and resided in the southern region of Saudi Arabia. The health practitioners in the sample represented 36.5%, and the remainder were non-health practitioners. Those with chronic diseases represented approximately 15% of the sample compared with healthy participants, who represented approximately 85% (Table 1).

In this sample, the prevalence of the SIV uptake during the preceding 12 months for the entire sample was 31.8%. Those aged 45–54 had the highest prevalence of SIV uptake (40%) compared with the other age groups. In addition, males had a much higher SIV uptake prevalence than females (38.9% vs. 21%). Region-wise, the western and southern regions had the highest SIV uptake, at 38.9% and 37.8%, respectively, compared with the other regions. Furthermore, health practitioners had a 41.5% prevalence of SIV uptake compared with non-health practitioners (26.2%). Similarly, the prevalence of SIV among those with chronic diseases was 44% compared with those without chronic diseases (29.6%) (Table 1).

**Figure 1:**
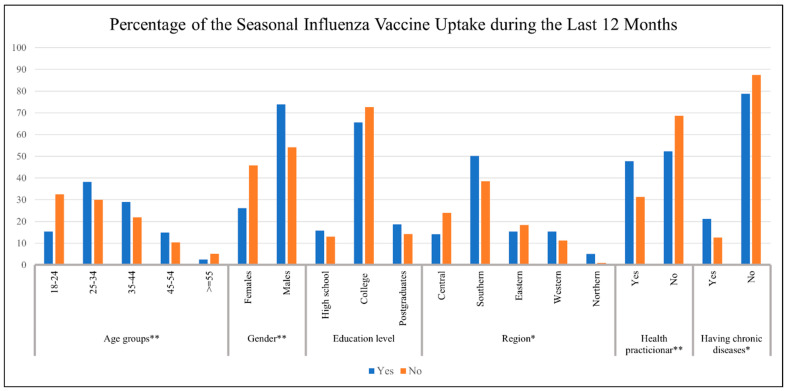
Percentage of the Seasonal Influenza Vaccine Uptake during the preceding 12 Months. * Significant with *p*-value < 0.01 based on the chi-squared distribution; ** Significant with *p*-value < 0.001 based on the chi-squared distribution.

The chi-squared results indicated that the SIV uptake during the preceding 12 months was significantly associated with age group (*p*-value < 0.001), gender (*p*-value 0.001), region (*p*-value = 0.001), being a health practitioner (*p*-value < 0.001), and having a chronic disease (*p*-value = 0.002). However, education level was not associated with the uptake of the SIV during the preceding 12 months (Table 2 and Figure 1).

Results from the logistic regression indicated that age was a significant predictor of the SIV uptake during the preceding 12 months. That is, the age groups that had higher odds of the SIV uptake during the preceding 12 months compared with those who were 55 years old or older were 25–34 (OR: 3.20; 95% CI: 1.14–9.00; *p*-value = 0.028), 35–44 (OR: 3.66; 95% CI: 1.33–10.05; *p*-value = 0.012), and 45–54 (OR: 3.29; 95% CI: 1.17–9.29; *p*-value = 0.025). Gender was also significantly associated with the uptake of the SIV during the preceding 12 months, as males had 73% higher odds of receiving the vaccine than females (OR: 1.73; 95% CI: 1.18–2.55; *p*-value = 0.005). Moreover, those who resided in the southern and western regions of Saudi Arabia had more than double the odds of having received the SIV during the preceding 12 months compared with the residents in the central region (OR: 2.16; 95% CI: 1.35–3.46; *p*-value = 0.001) and (OR: 2.40; 95% CI: 1.32–4.37; *p*-value = 0.004), respectively. Similarly, health practitioners were almost twice as likely to have received the SIV during the preceding 12 months as non-health practitioners (OR: 2.11; 95% CI: 1.45–3.06; *p*-value = 0.001). Finally, those with chronic diseases were 86% more likely to have received the SIV during the preceding 12 months than those without chronic diseases (OR: 1.86; 95% CI: 1.18–2.95; *p*-value = 0.008) (Table 3).

## 4. Discussion

The present study aimed to estimate the prevalence and predictors of seasonal influenza vaccine (SIV) uptake in Saudi Arabia. A relatively low prevalence of SIV uptake during the preceding 12 months in the Saudi population was found (31.8%). Lower SIV uptake was observed among those aged 55 years or older (18.2%), females (21%), residents of the central region (21.5%), non-health practitioners (26.2%), and those without chronic diseases (29.6%). Several factors were considered to be major predictors of the uptake of SIV in Saudi Arabia. These factors included age, gender, region, health practitioner status, and prevalent chronic diseases.

The prevalence of SIV uptake in this current study was relatively lower than those from other studies conducted among the Saudi population, where the prevalence ranged from 44% to 55% [27,28,35,36,37,38]. However, those studies were conducted prior to the COVID-19 pandemic (2017–2020), which may raise concerns about whether the COVID-19 era has played a role in SIV uptake hesitancy among the public. A recent study found that previous COVID-19 vaccination was negatively associated with the willingness to vaccinate against seasonal influenza due to the perceived fear of multiple COVID-19 doses and boosters taken during the pandemic [24]. Another study found that those who reported side effects from the COVID-19 vaccine were 13% less willing to take the SIV [25]. In addition, a recent study conducted in Saudi Arabia during the pandemic highlighted concerns regarding the potential interaction between SIV and the COVID-19 vaccine, in addition to concerns relating to the uptake of multiple COVID-19 boosters negatively affecting SIV uptake [29].

Another explanation of the observed discrepancies between previous studies in Saudi Arabia with a higher prevalence of reported SIV uptake may have been attributed to the outcome assessment. The prevalence of SIV uptake in the previous studies was measured based on whether the participants had ever been vaccinated against seasonal influenza [27,35,36,38]. In contrast, the main objective of our study was to measure whether the participant has received the SIV during the preceding 12 months. Moreover, another study conducted on those aged 65 years or older found a prevalence of SIV uptake of approximately 52% [28], which was different from the prevalence found in our study, mainly due to the small proportion of elderly people in our sample. Approximately 4.5% of individuals within our sample were aged 55 years or older. It is possible that they were perhaps younger than 65 and healthier, or had less perceived risk of being infected with influenza or suffering from influenza-related complications. 

Furthermore, we found that middle-aged groups had over three-fold higher odds of SIV uptake in Saudi Arabia than those aged 55 years or older. This can be attributed to policies and work requirements (mainly for middle-aged workers) who regularly receive direct recommendations from their employers to receive the vaccine in order to avoid absenteeism [15]. Furthermore, we found that males have higher odds of being vaccinated against seasonal influenza, which is consistent with previous studies from the Saudi population [27,28]. A previous study in Saudi Arabia explored the factors associated with lower SIV uptake rates among females than males. They found that females had expressed more fear of the SIV injections or vaccine-related side effects [39].

Regarding healthcare workers, we found that health practitioners had over two-fold higher odds of SIV uptake than non-health practitioners. Our findings were consistent with other studies where healthcare workers had a higher prevalence of SIV uptake than the general population [40,41]. We also observed that 41.5% of health practitioners had been vaccinated against seasonal influenza, which is slightly comparable with the research of Alsuhaibani and Awadalla et al., who found prevalences of 48.6% and 45.5%, respectively [40,41]. The perceived risk can mainly explain the higher prevalence of SIV uptake among healthcare workers who are likely to encounter the virus in healthcare facilities [42].

In addition, we noticed that those with chronic diseases had higher odds of receiving the SIV than those without chronic diseases, which is comparable with previous studies [43,44]. Although not significant due to the smaller sample size than ours, Alkathlan et al. found that those with comorbidities had 23% higher odds of being vaccinated against seasonal influenza than those without comorbidities [43]. Similarly, the prevalence of SIV uptake among diabetic patients was 61%, which is considerably higher than that of the general population [44].

Seasonal influenza can be associated with severe morbidity, which may lead to significant health complications and increased rates of absenteeism from work. However, the health and economic burden of seasonal influenza can be reduced by increased rates of SIV uptake in the population. Unlike other nations in the world who have expressed faith-based reasons against SIV uptake, the Saudi population (nearly 100% Muslim) has not expressed religious incompatibility with the vaccination, which may raise concerns regarding other potential factors associated with the low SIV uptake [45]. In a study conducted in Saudi Arabia before the COVID-19 pandemic, the misconception regarding vaccination was reported as vaccines having been introduced for commercial purposes [46]. Such a misconception could have been sustained during and post the COVID-19 pandemic for all vaccines, including the SIV.

The healthcare system in Saudi Arabia is managed and regulated by the Ministry of Health (MOH). The Saudi MOH is the government sector that proposes to the King of Saudi Arabia any economic, industrial, and social regulations and policies that should be used to mitigate potential health burden in the Kingdom. The MOH can utilize the results of this study to address the low vaccine uptake rate and the associated factors among the Saudi population. A specific focus should be on those aged 55 years or older and females who perhaps did not have job requirements or policies enforcing mandatory vaccination, as some private-sector employers in the Kingdom require SIV uptake for their employees to reduce the potential absence from work due to influenza and ILI. In terms of regional variation, MOH should enhance vaccination campaigns in regions with low uptake rates, particularly the central and northern regions. Furthermore, the observed lower uptake rate in this study, compared with previous studies conducted in Saudi Arabia, could have resulted from the post COVID-19 myths and misinformation concerning the vaccine. Misinformation may cause mistrust in the healthcare system [26]. A recent study in Saudi Arabia found that health misinformation significantly predicted a low acceptance rate of the COVID-19 vaccine during the pandemic [47]. The role of misinformation during the pandemic may have caused an increased level of hesitancy among those who usually receive the SIV on a regular basis. The spread of misinformation through social media needs to be faced with extraordinary campaigns conducted by the Saudi MOH to encourage SIV uptake, such as those undertaken during the COVID-19 pandemic. Official and extensive educational health programs utilizing social media and TV advertisements should be carried out. Additionally, new policies and regulations—such as those implemented during the COVID-19 pandemic—mandating SIV for travelling and entrance to public places should be considered, especially with the current increased rates of seasonal influenza cases and influenza-like illnesses. Such regulations have been effective and have led to the increased uptake of COVID-19 vaccination during the pandemic [48,49]. SIV is effective and safe with considerably mild side effects [50,51]; hence, regulations that mandate annual vaccine uptake may be safely implemented to reduce the seasonal influenza health and economic burden in Saudi Arabia. 

In the present study, we collected information from all five main provinces in Saudi Arabia with a sufficient sample size, which may have enhanced the study’s statistical power. However, several limitations to this study should be acknowledged, mainly related to the measurement tool used. First, the survey was not validated, which could have resulted in measurement error due to potential misclassification. However, this type of misclassification is non-differential, and it would only bring the measure of effect toward the null [52]. Furthermore, we have conducted thorough and extensive revisions and edits to improve the understanding of the questions and the survey choices and to shorten the response time to prevent potential invalid responses due to lack of care or boredom. Second, we have not collected information regarding nationality to assess whether Saudis had higher or lower uptake rates than non-Saudis. However, we have no reason to believe that nationality could have influenced the uptake rates because of the availability of the vaccine for Saudis and non-Saudis. Third, the survey was distributed in Arabic, which may have limited the generalizability of the results to those who clearly understand Arabic. Fourth, generalizability may have been affected by the use of the non-probability sampling technique (snowball), which may not have yielded a representative sample of the Saudi population. The age distribution of the participants in our study demonstrated insufficient representativeness as we had a relatively small proportion of the high-priority group to receive SIV (≥55 years)—possibly due to the lack of social media use among this particular group. Such an issue may have underestimated the prevalence in this study. In addition, generalizability may have been affected by the eligibility to participate in the study. That is, the participant should have had a smartphone and internet access in order to receive the survey link; hence, those who did not use smartphones were not included in the sampling frame. However, recent data from the General Authority for Statistics in Saudi Arabia have reported that over 90% of the Saudi population possessed smartphones and had access to the internet [53]. Despite these issues with generalizability, the findings are still considered to be relevant to the population under study. 

## 5. Conclusions

This study investigated the prevalence of seasonal influenza vaccine (SIV) uptake during a 12-month period (post the COVID-19 pandemic) in Saudi Arabia. In general, there was a considerably low SIV uptake in the studied population, especially among those aged 55 years or older, females, residents of the central region, non-health practitioners, and those without chronic diseases. Although these results should be cautiously interpreted and more sophisticated studies are needed to assess the SIV uptake rates post the COVID-19 pandemic, the findings provide insights into the possibility of a low prevalence and the predictors of SIV uptake in Saudi Arabia post the COVID-19 pandemic. Therefore, the Saudi MOH should develop and implement effective strategies and recommendations—such as those implemented during the COVID-19 pandemic—including reinforcing policies and regulations to increase the SIV uptake rates in Saudi Arabia. Such policies may eventually mitigate the health and economic burdens of seasonal influenza in society.

## Figures and Tables

**Table 1:** Characteristics of the respondents and prevalence of the seasonal influenza vaccine (SIV) uptake during the preceding 12 months per group (N = 758).

Variable	Respondents		SIV Uptake Prevalence per Group	
	n	(%)	n	(%)
**Age group**				
18–24	205	(27.0)	37	(18.0)
25–34	247	(32.6)	92	(37.2)
35–44	183	(24.1)	70	(38.3)
45–54	90	(11.9)	36	(40.0)
≥55	33	(4.4)	6	(18.2)
**Gender**				
Female	300	(39.6)	63	(21.0)
Male	458	(60.4)	178	(38.9)
**Education level**				
High school or less	105	(13.9)	38	(36.2)
Bachelor’s degree or some college education	534	(70.4)	158	(29.6)
Postgraduate	119	(15.7)	45	(37.8)
**Region**				
Central	158	(20.8)	34	(21.5)
Southern	320	(42.2)	121	(37.8)
Eastern	132	(17.4)	37	(28.0)
Western	95	(12.5)	37	(38.9)
Northern	53	(7.0)	12	(22.6)
**Health practitioner**				
Yes	277	(36.5)	115	(41.5)
No	481	(63.5)	126	(26.2)
**Has chronic diseases**				
Yes	116	(15.3)	51	(44.0)
No	642	(84.7)	190	(29.6)

**Table 2:** Bivariate analysis of the associations between multiple variables and seasonal influenza vaccine uptake.

Variable	Seasonal Influenza Vaccine Uptake during the Preceding 12 Months		*p*-Value *
	Yes	No	
	241 (31.8%)	517 (68.2%)	
**Age group, n (%)**			<0.001
18–24	37 (15.4)	168 (32.5)	
25–34	92 (38.2)	155 (30.0)	
35–44	70 (29.0)	113 (21.9)	
45–54	36 (14.9)	54 (10.4)	
≥55	6 (2.5)	27 (5.2)	
**Gender, n (%)**			<0.001
Female	63 (26.1)	237 (45.8)	
Male	178 (73.9)	280 (54.2)	
**Education level, n (%)**			0.127
High school or less	38 (15.8)	67 (13)	
Bachelor’s degree or some college education	158 (65.6)	376 (72.7)	
Postgraduate	45 (18.7)	74 (14.3)	
**Region, n (%)**			0.001
Central	34 (14.1)	124 (24.0)	
Southern	121 (50.2)	199 (38.5)	
Eastern	37 (15.4)	95 (18.4)	
Western	37 (15.4)	58 (11.2)	
Northern	12 (5.0)	41 (7.9)	
**Health practitioner, n (%)**			<0.001
Yes	115 (47.7)	162 (31.3)	
No	126 (52.3)	355 (68.7)	
**Has chronic diseases, n (%)**			0.002
Yes	51 (21.2)	65 (12.6)	
No	190 (78.8)	452 (87.4)	

* Derived from chi-squared distribution.

**Table 3:** Predictors of seasonal influenza vaccine uptake *.

Variable	Having Received the Seasonal Influenza Vaccine during the Preceding 12 Months	
	OR (95 %CI)	*p*-Value
**Age group**		
18–24	1.34 (0.46–3.89)	0.589
25–34	3.20 (1.14–9.00)	0.028
35–44	3.66 (1.33–10.05)	0.012
45–54	3.29 (1.17–9.29)	0.025
≥55	Ref	
**Gender**		
Female	Ref	
Male	1.73 (1.18–2.55)	0.005
**Education level**		
High school or less	1.78 (0.95–3.36)	0.073
Bachelor’s degree or some college education	1.06 (0.67–1.67)	0.805
Postgraduate	Ref	
**Region**		
Central	Ref	
Southern	2.16 (1.35–3.46)	0.001
Eastern	1.45 (0.82–2.54)	0.198
Western	2.40 (1.32–4.37)	0.004
Northern	1.01 (0.46–2.21)	0.990
**Health practitioner**		
Yes	2.11 (1.45–3.06)	<0.001
No	Ref	
**Has chronic diseases**		
Yes	1.86 (1.18–2.95)	0.008
No	Ref	

* Derived from binary logistic regression.

## Data Availability

The data are available from the corresponding author upon reasonable request.
